# Rapid and Efficient Extraction of Chlorogenic Acid from Honeysuckle Using Microwave-Assisted Deep Eutectic Solvents: Process Optimization and Mechanistic Investigation via Integration of Experiments and Density Functional Theory Calculations

**DOI:** 10.3390/molecules31193545

**Published:** 2026-10-04

**Authors:** Hongwei Wu, Ruixin Chen, Hu Feng, Ningfei Liu, Yongli Shi, Feng Wang, Tiancheng Mu

**Affiliations:** 1 School of Basic Medical Sciences, Henan Medical University, Xinxiang 453003, China; 18338918237@163.com (R.C.); fh20190617@163.com (H.F.); 13849924802@163.com (N.L.); 2 College of Pharmacy, Henan Medical University, Xinxiang 453003, China; shiyongli2005@163.com; 3 Henan Key Laboratory of Tissue Regeneration, School of Basic Medical Sciences, Henan Medical University, Xinxiang 453003, China; fengw@xxmu.edu.cn; 4 School of Chemistry and Life Resources, Renmin University of China, Beijing 100872, China

**Keywords:** chlorogenic acid (CA), deep eutectic solvent (DES), microwave-assisted extraction (MAE), response surface methodology (RSM), density functional theory (DFT), honeysuckle

## Abstract

Chlorogenic acid (CA), the principal bioactive constituent of honeysuckle, is conventionally extracted by time-consuming methods using toxic organic solvents, which carry the risk of thermal degradation. This study provides a rapid and efficient microwave-assisted deep eutectic solvent (DES-MAE) method for CA extraction and elucidates its mechanism at the molecular level. Eight DESs were systematically screened, and extraction parameters were optimized via single-factor experiments and response surface methodology (RSM). Density functional theory (DFT) calculations were performed to reveal the DES-CA interaction mechanism. Benzyltrimethylammonium chloride–ethylene glycol with 40% water content was identified as optimal. Under the optimized conditions (640 W, 78 s, and 9.35 mg/mL), the quadratic model (*R*^2^ = 0.9879) predicted a CA yield of 39.851 mg/g, which was consistent with the experimental value of 39.431 ± 0.068 mg/g (1.1% relative deviation). DFT calculations revealed four hydrogen bonds and π-π stacking between DES1 and CA, with a binding energy of −25.641 kcal/mol, which far exceeded those of water (−5.277 kcal/mol) and ethanol (−8.946 kcal/mol). The DES-MAE method achieved the highest extraction yield within merely 78 s, compared with 60 min for HRE and 30 min for the pharmacopoeial method. This work provides a rapid, efficient, and mechanistically validated strategy for extracting bioactive compounds from traditional Chinese medicinal materials.

## 1. Introduction

Honeysuckle, an important medicine–food homologous plant, is widely cultivated across East Asia, including China, Japan and the Republic of Korea [1]. Notably, in China, the large-scale cultivation of honeysuckle in Fengqiu County of Henan Province can be traced back 1500 years. As a core heat-clearing and detoxifying herb, honeysuckle is administered either as a single agent or as a pivotal constituent in numerous traditional Chinese medicinal prescriptions, with well-documented clinical applications for the treatment of exogenous wind–heat syndrome, epidemic febrile diseases, sores, carbuncles, furuncles, and a broad spectrum of infectious conditions [2]. Modern pharmacological studies have validated that the therapeutic efficacy of honeysuckle primarily stems from its abundant bioactive constituents, including phenolic acids, flavonoids, polysaccharides, and essential oils [3,4]. Among these active ingredients, chlorogenic acid (CA), a representative caffeoylquinic acid derivative, has been officially adopted as the quality control marker for honeysuckle in the Chinese Pharmacopoeia and is recognized as an important bioactive constituent responsible for the herb’s pharmacological effects. Accumulating evidence has demonstrated that CA exerts broad-spectrum antibacterial activity by disrupting bacterial membrane integrity and inhibiting biofilm formation [5,6]. Furthermore, CA exhibits versatile biological activities, such as antiviral, antitumor, and glucolipid metabolic-regulating properties, rendering it a high-value natural product with a continuously growing market demand [7]. Therefore, the development of innovative rapid and efficient extraction strategies to enhance CA extraction yield is of great importance for the high-value utilization of honeysuckle medicinal resources.

To date, various techniques have been employed to extract CA from honeysuckle. Among them, conventional heat-reflux extraction (HRE) is simple and low-cost but time-consuming, typically requiring hours of operation and large amounts of organic solvents, while prolonged heating risks the thermal degradation of the heat-labile CA [8]. Therefore, instrument-assisted extraction methods for CA, such as ultrasonic-assisted extraction (UAE) [9,10,11] and microwave-assisted extraction (MAE) [12], have attracted increasing attention owing to their higher extraction efficiency, faster extraction process, and lower solvent consumption. Compared with UAE, MAE can generate volumetric dielectric heating, which rapidly disrupts plant cells and accelerates the mass transfer process, thus generally resulting in shorter extraction times and higher extraction efficiency [13]. However, conventional MAE of CA from honeysuckle relies on volatile and toxic organic solvents such as ethanol as the extraction medium, which raises environmental and safety concerns and is inconsistent with the increasing demand for rapid and sustainable extraction processes [8,12]. These drawbacks motivate the search for alternative, greener extraction solvents that can be combined with the benefits of microwave irradiation.

Deep eutectic solvents (DESs), formed by combining a hydrogen-bond acceptor (HBA) with a hydrogen-bond donor (HBD), have emerged as a promising class of green solvents [14,15]. Compared with conventional organic solvents and ionic liquids, DESs are inexpensive, easy to prepare, biodegradable, and exhibit low toxicity and negligible volatility, while their physicochemical properties can be readily tailored by adjusting the type and molar ratio of their components [16,17]. More importantly, DESs can disrupt the plant cell-wall structure by solubilizing lignin, a key barrier that encapsulates intracellular bioactive compounds. Moreover, as polarity-tunable solvents, DESs can efficiently absorb microwave energy. Thus, the integration of DESs with MAE provides an efficient approach for the extraction of bioactive constituents from plants [18,19]. It has been reported that coupling a choline chloride-based DES with MAE afforded higher yields of bioactive compounds from cocoa bean shell than DES extraction alone [20]. Olalere et al. found that DES-MAE recovered 33.0% of the protein from defatted wheat germ in merely 3 min and 28 s without any detectable protein hydrolysis or degradation, in contrast to conventional alkaline extraction, which needed 1 h and yielded only 12.5% [21]. Jin et al. developed an MAE-DES method for extracting anthocyanins from *purple-heart radish.* Benefiting from the synergistic effect between the microwave field and DES, the DES-MAE method was significantly superior to HRE, UAE, and MAE using an acidic ethanol solution as the extractant [22]. Furthermore, Zhu et al. applied the DES-MAE method to the extraction of total phenolic content from walnut green husks. By comparing the extraction performance of DES-MAE, MAE coupled with water or ethanol, and DES, it was found that DES-MAE afforded the highest total phenolic content within only 4 min [23]. As can be seen from the literature, conventional DES extraction without microwave assistance typically requires long processing times (30–60 min) owing to the high viscosity of DESs and slow thermal diffusion. Although UAE enhances cell-wall disruption through cavitation effects, its processing time remains relatively long (typically 20–30 min), and its energy utilization is less direct than that of microwave heating. In contrast, the DES-MAE approach integrates the strong solubilizing capacity of DESs with the rapid volumetric heating and cell-wall-disrupting effect of microwaves, thereby representing a highly promising extraction strategy. To the best of our knowledge, the systematic combination of experimental and molecular-simulation methods for investigating the microwave-assisted extraction of CA from honeysuckle using deep eutectic solvents has not been reported so far.

In the present study, a rapid and efficient extraction method integrating microwave-assisted deep eutectic solvent extraction with HPLC was developed for the isolation of CA from honeysuckle. Key operating variables governing the extraction efficiency were first investigated through single-factor experiments, and the optimal extraction conditions were subsequently determined by employing the Box–Behnken design (BBD). To gain insight into the molecular mechanism underlying the extraction process, DFT calculations were performed in conjunction with experimental observations. In addition, the proposed approach was benchmarked against conventional heat-reflux extraction and the pharmacopoeial method. Overall, this work offers an efficient and sustainable alternative for CA recovery from honeysuckle and supports the high-value utilization of bioactive constituents in traditional Chinese medicinal materials.

## 2. Results and Discussion

### 2.1. Optimization of DES-MAE

#### 2.1.1. Selection of DES Type

**Figure 1:**
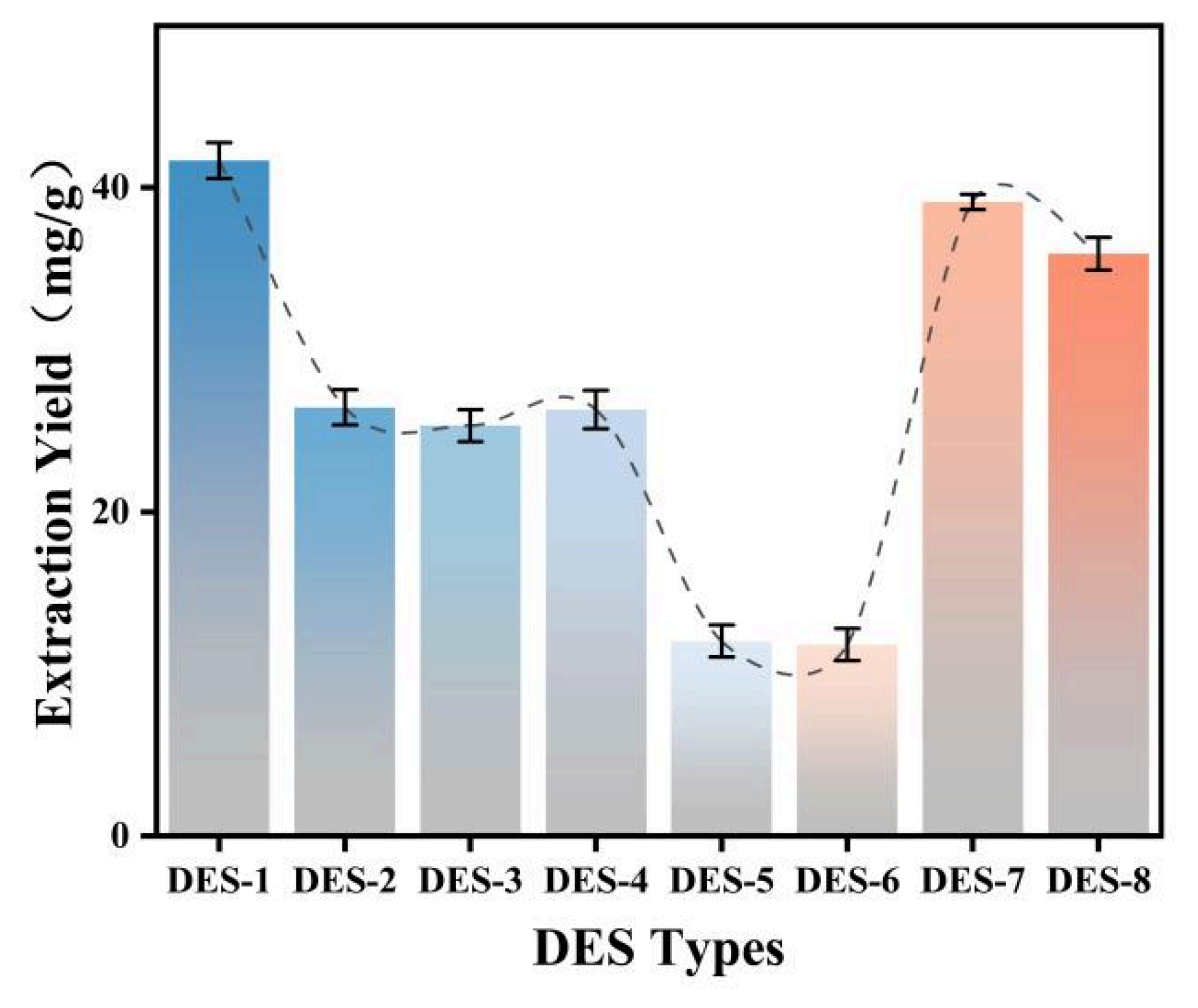
Effect of DES types on extraction yield of CA.

**Figure 2:**
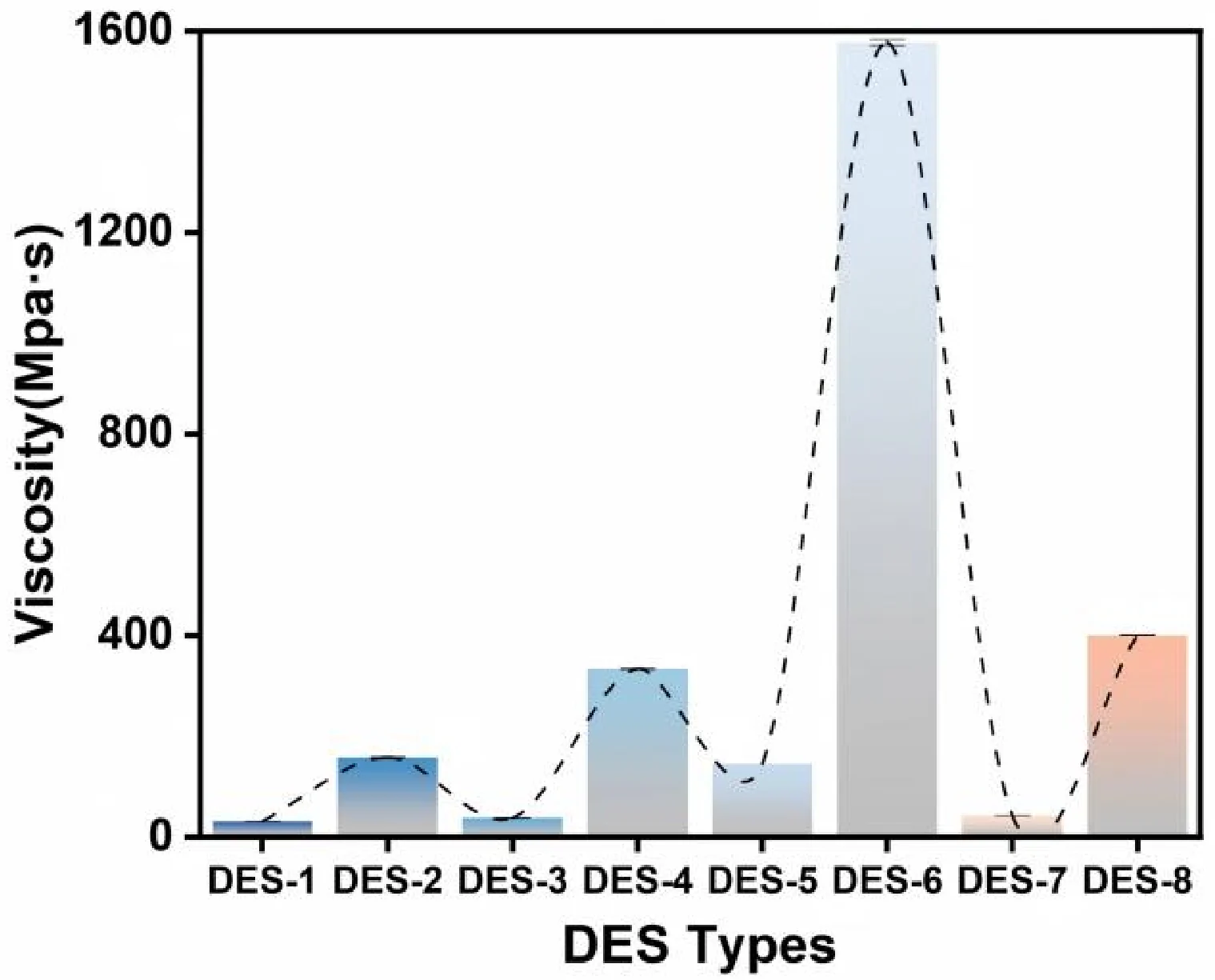
Viscosity of different DES types.

DESs are gaining popularity as greener substitutes for traditional organic solvents owing to a suite of characteristics consistent with the advancement of green chemistry. The type of DES plays a crucial role in the efficient extraction of active constituents from herbal plants [24]. Building upon our previous work, eight DESs were selected in this study for the microwave-assisted extraction of CA from honeysuckle. The results shown in Figure 1 indicate that the extraction ability of different DESs follows the order: DES1 > DES7 > DES8 > DES2 > DES4 > DES3 > DES5 > DES6. According to the literature [25], the DES extraction process is influenced by multiple interactions, including hydrophobic interactions, hydrogen bonding, and π-π interactions. Meanwhile, the viscosity and polarity of the DES also affect the extraction efficiency. For DESs with the same HBA, the extraction yields of CA obtained with DESs containing ethylene glycol (EG) as the HBD were higher than those obtained with DESs containing glycerol (Gly) as the HBD. This can be explained from the following perspectives: although Gly possesses more hydroxyl groups than EG and can thus more readily form hydrogen bonds with CA, the three hydroxyl groups in Gly readily form intramolecular hydrogen bonds, making it more prone to self-association within the DES system, thereby reducing the effective sites available for hydrogen bonding with CA [26]. Meanwhile, viscosity measurements of the eight DESs using a rotary viscometer (model NDJ-8S, Changji Geological Instrument Co., Ltd., Shanghai, China) at 25 °C demonstrated that the viscosities of Gly-based DESs were significantly higher than those of EG-based DESs (Figure 2). The high viscosity of DESs impedes solute diffusion and mass transfer, thereby reducing the extraction efficiency. For DESs with the same HBD, the extraction yield of CA obtained with DESs employing benzyltrimethylammonium chloride (TMBAC) as the HBA was higher than those obtained with DESs using benzyltriethylammonium chloride (TEBAC) or benzyltributylammonium chloride (TBBAC) as the HBA. This can be mainly attributed to the fact that the CA molecule contains one strongly polar carboxyl group and six hydroxyl groups, endowing it with high polarity and good water solubility. For benzylammonium chloride-based DESs with different alkyl chain lengths, the hydrophobicity increases with increasing alkyl chain length. Therefore, according to the “like dissolves like” principle, TMBAC-based DESs, which possess the lowest hydrophobicity, exhibit the strongest hydrophobic interaction with CA molecules. The high extraction yields of DES7 and DES8 are mainly ascribed to the absence of a benzyl group in the tetrabutylammonium chloride (TBAC) structure, which results in less steric shielding and allows Cl^−^ in the molecule to act as an HBA, forming effective hydrogen bonds with the hydroxyl and carboxyl groups of CA. Meanwhile, DES7 and DES8 also exhibit lower viscosities, thereby contributing to higher extraction efficiencies. In summary, multiple interactions are involved in the extraction of CA, and these interactions exert different effects on the extraction performance. According to the experimental results, DES1 exhibited the highest extraction efficiency due to its lowest viscosity, strongest polarity, effective hydrogen bonding with CA, and the presence of π-π interactions between DES1 and CA molecules. Accordingly, DES1 was selected as the extraction solvent for the subsequent optimization experiments. It should be clarified that DES1 refers to the binary DES composed of TMBAC and EG at a molar ratio of 1:1. The actual extraction solvent used in the experiments was the DES1–water mixture containing 40% (*w*/*w*) water, which was prepared by adding deionized water to the binary DES1. For DFT calculations, only the binary DES1 (TMBAC–EG) without explicit water molecules was modeled to elucidate the intrinsic intermolecular interactions between DES1 and CA.

#### 2.1.2. Water Content

**Figure 3:**
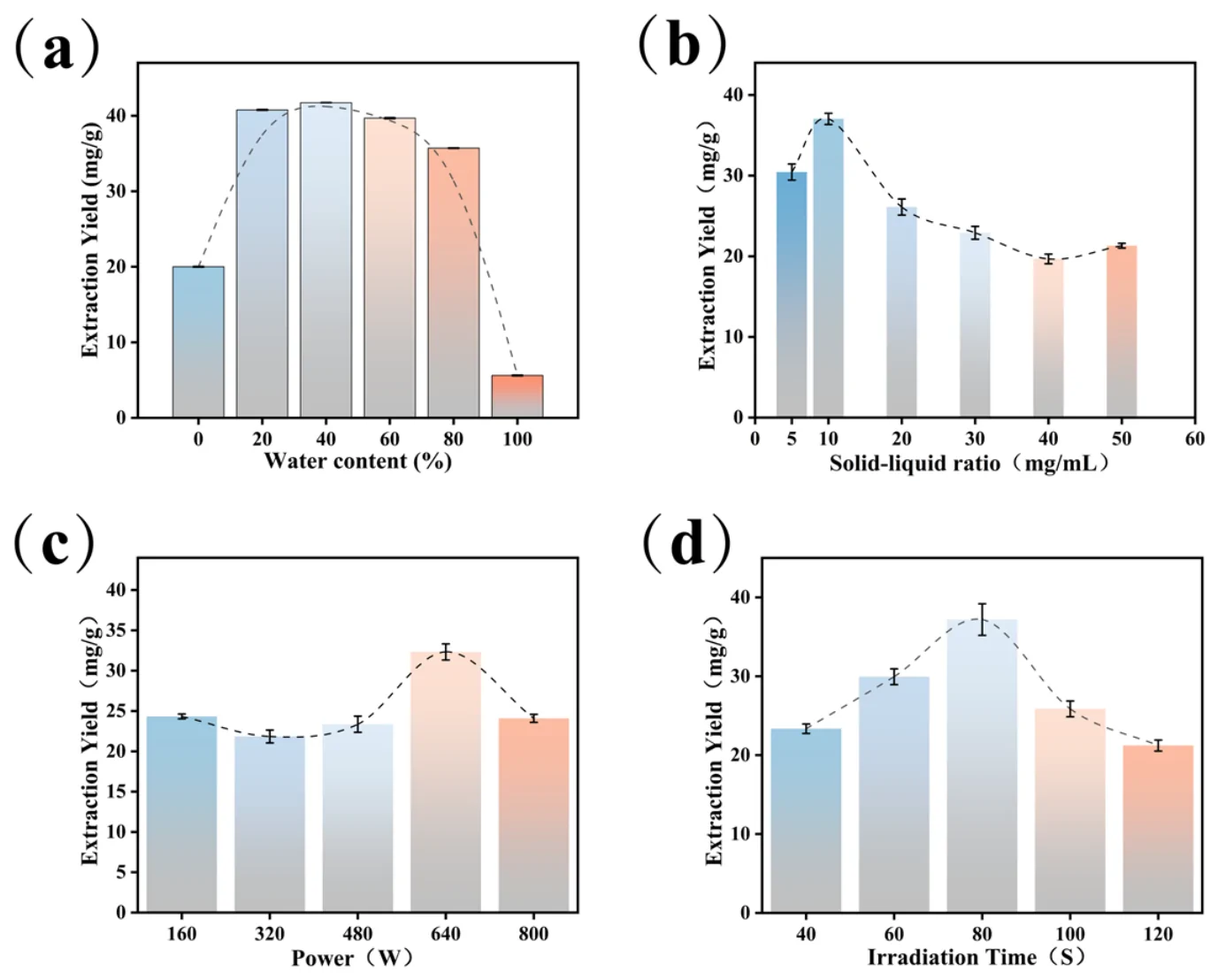
Effect of water content (**a**), solid–liquid ratio (**b**), microwave power (**c**) and irradiation time (**d**) on the extraction of CA under the optimal conditions.

The addition of an appropriate amount of water to a DES can alter its viscosity and polarity, thereby enhancing the mass transfer of the target analyte between the solid and liquid phases and ultimately affecting the extraction efficiency. Therefore, the effect of DES–water content on the extraction yield of CA was investigated under optimized conditions. As shown in Figure 3a, the extraction yield of CA gradually increased as the water content of the DES increased from 0% to 40%, whereas it began to decline when the water content exceeded 40%. This can be mainly explained as follows: at a water content of 0%, the neat DES could not be uniformly mixed with the sample, and the mass transfer of CA between the solid and liquid phases was also hindered. The addition of water reduced the viscosity of the DES extraction system, enabling uniform mixing with the sample. Meanwhile, the presence of water caused the sample matrix to swell, increasing its contact area with the extractant and thus leading to an improved extraction yield. However, when the water content exceeded 40%, the excessive water disrupted the hydrogen-bonding network of the DES, weakening the interactions between the DES and CA, which was unfavorable for CA extraction [27]. It is particularly noteworthy that the extraction efficiency was the lowest when pure water was used as the extractant, confirming the significant role of DES in the extraction process. This may be attributed to the fact that, in addition to the multiple interactions between DES and CA, DES also exerts a strong disruptive effect on plant cell walls. Accordingly, a DES–water content of 40% was ultimately selected for subsequent experiments.

#### 2.1.3. Solid–Liquid Ratio

The solid–liquid ratio plays a crucial role in the extraction efficiency of natural products. Therefore, the effect of different solid–liquid ratios on the extraction efficiency of CA from honeysuckle was investigated. In this study, the solid-to-liquid ratio was defined as the mass of honeysuckle powder (mg) divided by the volume of DES containing 40% water content (mL). As shown in Figure 3b, the extraction yield of CA increased as the solid–liquid ratio increased from 5 mg/mL to 10 mg/mL, and the highest CA extraction yield was obtained at a solid-to-liquid ratio of 10 mg/mL. This indicates that the amount of extractant was sufficient to completely extract CA from honeysuckle at this ratio. However, further increasing the solid–liquid ratio may lead to an insufficient amount of extractant, preventing it from mixing thoroughly with the target analytes and thereby reducing the CA extraction yield. Accordingly, to achieve the optimal extraction performance, a solid–liquid ratio of 10 mg/mL was selected for all subsequent experiments.

#### 2.1.4. Microwave Power

MAE rapidly heats the solvent and plant material via microwave radiation. Microwaves induce the rotation and movement of polar molecules and ions within the mixture, thereby generating internal heat. This rapid temperature rise leads to localized pressure accumulation within plant cells, promoting cell rupture and the release of intracellular contents. DESs possess an ionic character and abundant polar groups, such as hydroxyl and carboxyl groups, endowing them with a high dielectric constant and efficient microwave energy absorption [28]. When combined with microwave heating, the solubility and extraction rate of CA are expected to be significantly enhanced, thereby yielding a higher content of the target compound.

Since the magnitude of microwave energy can affect the interactions between the solvent and target compound molecules, microwave power is one of the critical factors influencing the final extraction efficiency. As shown in Figure 3c, the extraction yield of CA gradually increased to a maximum as the microwave power was raised from 160 W to 640 W, whereas it declined upon further increases in the microwave power. The primary reason may be that increasing the microwave power intensifies the coupling between microwave energy and the molecules in the extraction reaction, thereby leading to rapid heating of the extraction system, elevating the solvent temperature, and improving the solubility of the solute in the extraction medium. This facilitates the rapid dissolution of the target analytes and consequently enhances the extraction efficiency. At a microwave power of 640 W, CA could be completely extracted within 80 s. However, when the microwave power was further increased to 800 W, charring of the sample was observed after extraction, accompanied by a decrease in the extraction yield of CA. This was attributed to the high temperature induced by excessive microwave power. Accordingly, a microwave power of 640 W was ultimately selected for the experiments.

#### 2.1.5. Irradiation Time

As shown in Figure 3d, the microwave irradiation time is also an important parameter affecting the extraction efficiency. The extraction efficiency of CA increased as the microwave irradiation time was extended from 40 s to 80 s, reaching a maximum at 80 s. Upon further prolongation of the microwave irradiation time, the extraction efficiency tended to decline, which may be attributed to the degradation of CA caused by prolonged microwave heating. Therefore, 80 s was determined as the optimal microwave irradiation time. It is noteworthy that the extraction times of CA from honeysuckle by ethanol-based MAE [12], ionic liquid-based UAE [11], DES-based HRE [29] and DES-based UAE [10] typically range from 20 min to 2 h, and the extraction yields of CA by these methods are all lower than that achieved by the present method. Compared with the aforementioned reported methods, rapid and efficient extraction represents one of the advantages of the DES-based microwave-assisted extraction proposed in this study.

### 2.2. RSM Optimization

Based on the results of the single-factor experiments, RSM was further employed to evaluate the effects of the variables on the extraction efficiencies of CA. A total of 17 experiments were conducted according to the Box–Behnken design (Table 1). To investigate the relationship between the independent variables and the response (yields of CA), a second-order polynomial equation was applied to fit the experimental data, and the resulting model equation is presented as follows:

Y = −244.57 + 0.4574A + 3.249B + 2.698C − 0.0004905AB − 0.0003712AC + 0.01858BC − 0.0003283A^2^ − 0.01989B^2^ − 0.2098C^2^

where A–C are the microwave power, irradiation time and solid–liquid ratio, respectively.

Analysis of variance (ANOVA) was employed to evaluate the statistical significance of the established model, and the results are summarized in Table 2. As shown in Table 2, the model was highly significant (*F* = 63.73, *p* < 0.0001), while the lack of fit was not significant (*p* = 0.1826 > 0.05), indicating that the model was well-fitted and could accurately predict the variation in CA extraction yield. In addition, for the response surface model of CA extraction yield, the coefficient of determination (*R*^2^) of the model was 0.9879, the adjusted coefficient of determination (Adj *R*^2^) was 0.9724, and the predicted coefficient of determination (Pred *R*^2^) was 0.8650. The difference between Adj *R*^2^ and Pred *R*^2^ was less than 0.2, demonstrating good agreement between the predicted and experimental values. The coefficient of variation (*C.V.*) was 4.03% (<10%), indicating good experimental reproducibility [30]. The signal-to-noise ratio (precision) was 22.03 (>4), further confirming that the model possessed sufficient precision and reliability.

Furthermore, residual diagnostic analysis was performed to verify the adequacy of the model. As shown in Figure S1, the points in the normal probability plot of residuals approximately followed a straight line, indicating that the residuals were normally distributed. As can be seen from Figure S2, the residuals were randomly scattered with respect to the predicted values, without any obvious trend or funnel-shaped distribution, indicating homogeneity of variance. These results confirmed that the statistical assumptions of the model were satisfied, and the established quadratic regression model was reliable and could be used to predict the extraction yield of CA from honeysuckle.

**Figure 4:**
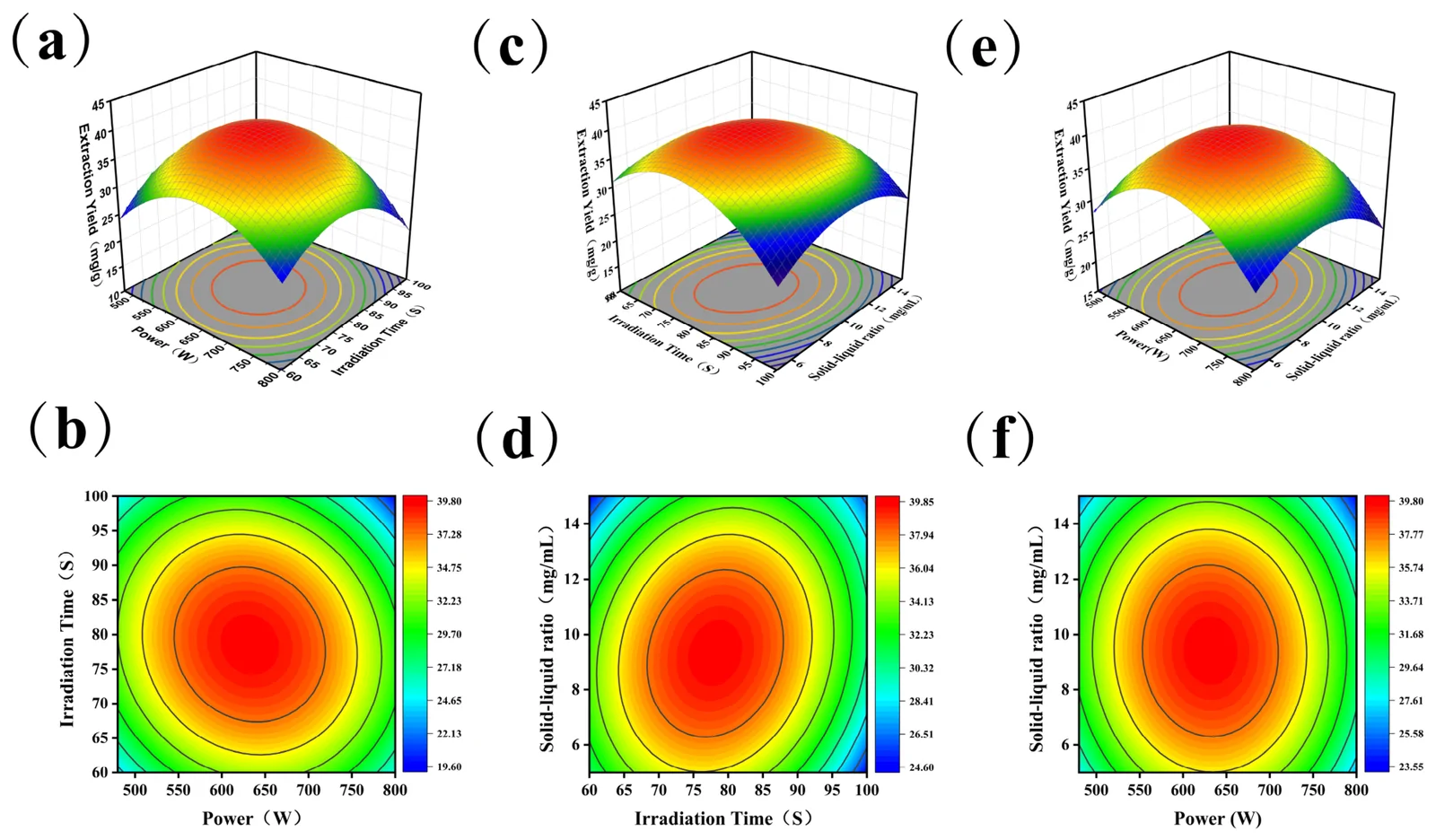
Three-dimensional (3D) response surface and contour plots of the interaction effects of different parameters on CA yield. 3D response surface and contour plots showing the effects of: (**a**,**b**) microwave power-irradiation time; (**c**,**d**) irradiation time-solid–liquid ratio; (**e**,**f**) microwave power-solid–liquid ratio.

The significance of the effects of each factor on CA extraction yield was as follows. The linear terms of the solid-to-liquid ratio and microwave time reached a significant level (*p* < 0.05), whereas the linear term of microwave power did not achieve significance (*p* = 0.06275). However, its quadratic term A^2^ was highly significant (*p* < 0.001), indicating that the effect of microwave power on extraction yield was primarily manifested as a nonlinear effect, i.e., the extraction yield followed a parabolic trend of first increasing and then decreasing with increasing power, which was consistent with the results of the single-factor experiments. The quadratic terms A^2^, B^2^, and C^2^ all reached a highly significant level (*p* < 0.001), and the sum of squares contributed by the quadratic terms accounted for 83.8% of the total model sum of squares, suggesting that the relationship between CA extraction yield and the three factors was predominantly nonlinear, with a well-defined optimal extremum point. Regarding the interaction effects, the interactions of AB (microwave power-irradiation time) and BC (irradiation time-solid–liquid ratio) were significant (*p* < 0.05), whereas the interaction of AC (microwave power-solid–liquid ratio) was not significant (*p* = 0.6328), indicating that irradiation time played a more important role in the synergistic effects with other factors. The interactive effects of parameters A, B, and C on CA extraction from honeysuckle were also visually presented in the three-dimensional response surface plots and contour plots of the model, as shown in Figure 4.

The aforementioned three factors were analyzed using Design-Expert 13, and the optimal extraction conditions were determined as follows: microwave power of 632.9 W, extraction time of 78.23 s, and solid-to-liquid ratio of 9.334 mg/mL. Under these optimal conditions, the predicted CA extraction yield was 39.851 mg/g. The 95% prediction interval for the CA yield at the predicted optimum was 39.851 ± 1.240 mg/g. To ensure experimental feasibility, the optimal conditions were adjusted as follows: microwave power of 640 W, extraction time of 78 s, and solid-to-liquid ratio of 9.35 mg/mL. Using these adjusted conditions, the experiment was repeated in triplicate. The CA extraction yield from honeysuckle was 39.431 ± 0.068 mg/g, with a relative deviation of 1.1%, which was in good agreement with the corresponding theoretical value. The weighing error (±0.1 mg, ±0.2%) and pipetting error (±0.5%, ±0.05 mg/mL) were within acceptable limits for routine laboratory practice. The minor deviation between the predicted and experimental optima was attributed to the rounding of operating parameters and the inherent variability of the extraction process.

### 2.3. Molecular Mechanism

#### 2.3.1. Structures and ESP of DES and DES-CA Complexes

**Figure 5:**
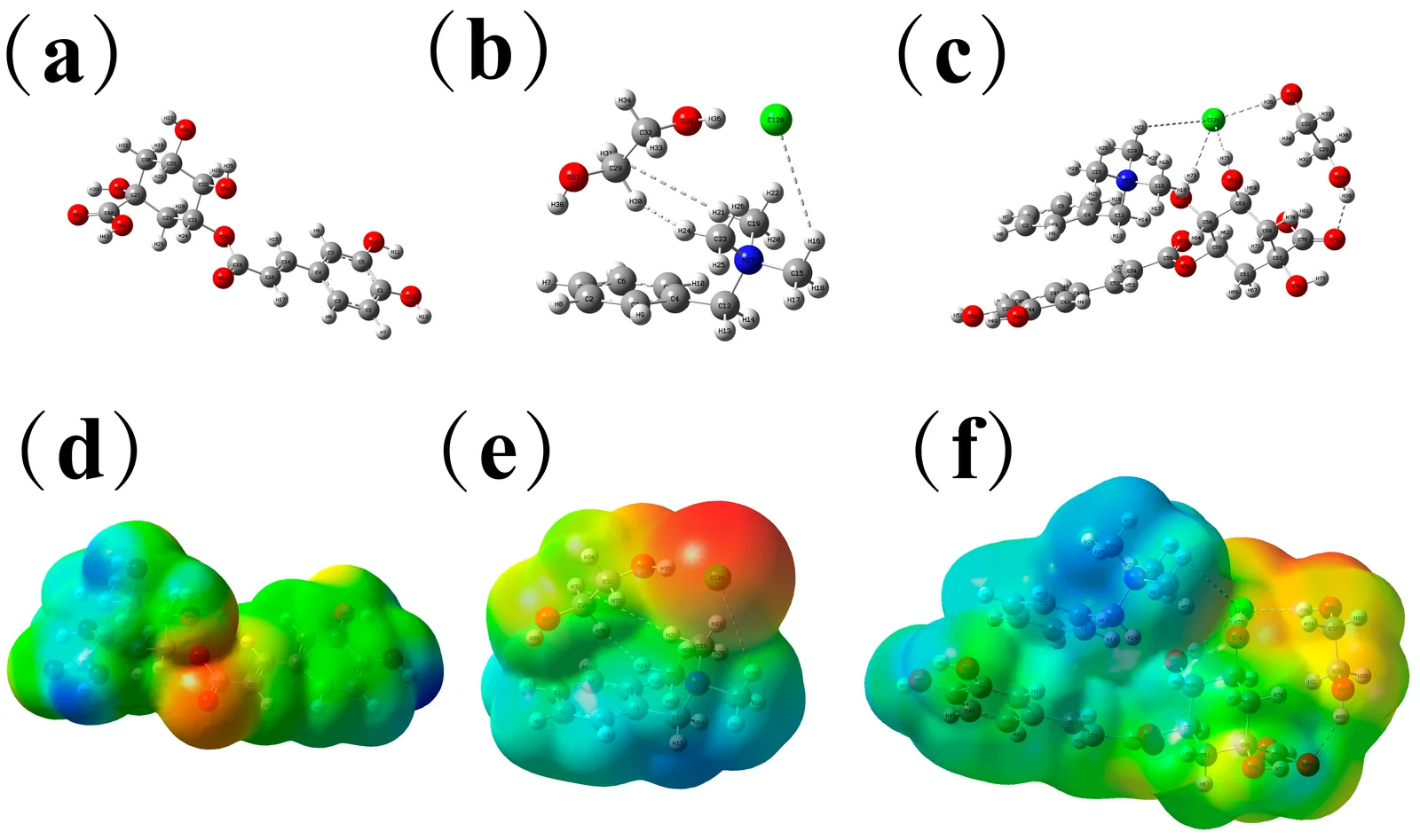
The structures and ESP of DES1, CA and the DES1-CA complex. Structures and ESP of CA (**a**,**d**); DES1 (**b**,**e**); DES1-CA complex (**c**,**f**).

Electrostatic potential (ESP) refers to the electrostatic potential energy at each atomic position in a molecular structure, which reflects the intramolecular charge distribution. The calculation of electrostatic potential is an important tool for delineating potential binding sites within a molecular system [31]. In the ESP maps, the color scheme adopted was as follows: blue represents positively charged regions, while red represents negatively charged regions. The intensity of these colors corresponds to the magnitude of the positive or negative charge, respectively. Figure 5 presents the ESP and structures of DES1, CA and the DES1-CA complex. DES1 was composed of one molecule of TMBAC and one molecule of EG. In TMBAC, the Cl^−^ anion exhibited the highest negative electrostatic potential (red), whereas the H atoms in the methyl and benzene rings, as well as the N atom, possessed positive electrostatic potentials (blue). In EG, the H atom of the -OH group exhibited a positive electrostatic potential (blue), while the O atom exhibited a negative electrostatic potential (red). Therefore, the formation of the TMBAC-EG complex may involve the interaction between the H atom of the -OH group in EG and the Cl^−^ anion in TMBAC. Following geometry optimization and single-point energy calculations, the theoretical configuration with the lowest energy and highest stability obtained from the TMBAC-EG calculations was the structure in which a hydrogen bond was formed between the Cl^−^ of TMBAC and the H atom of the -OH group in EG (Figure 5b).

For CA, the positive charges were mainly distributed on the H atoms of the -OH groups attached to the benzene ring and the cyclohexane ring, whereas the negative charges were primarily located on the O atom of the C=O group in the ester bond, as well as the O atoms of the -OH and -COOH groups on the cyclohexane and benzene rings (Figure 5a). Similar to the formation process of TMBAC-EG, the -OH groups on the benzene and cyclohexane rings of CA may form hydrogen bonds with the Cl^−^ anion in TMBAC. Meanwhile, the H atoms of the -OH groups in EG may form hydrogen bonds with the O atoms of the -COOH group on the cyclohexane ring and the C=O group of the ester bond in CA. Furthermore, the benzene ring of CA and the benzene ring of TMBAC may associate via π-π stacking interactions. Based on the calculations, the structure that best matched the expected configuration was the one in which the Cl^−^ of TMBAC formed hydrogen bonds with the H atoms of the -OH groups on both EG and the cyclohexane ring of CA, the -OH group of EG simultaneously formed a hydrogen bond with the O atom of the -COOH group on the cyclohexane ring of CA, and the benzene rings of TMBAC and CA were potentially associated via π-π stacking interactions (Figure 5c).

#### 2.3.2. Hydrogen Bond and Energies of DES and DES-CA Complexes

In general, hydrogen bond formation occurs between the H atom of an -OH or -NH_2_ group and another highly electronegative atom (e.g., N, O, Cl), and the bonding parameters (bond length and bond angle) should satisfy the standard hydrogen-bonding criteria (1.5 Å < L < 3.5 Å, 90° < θ < 180°) [32]. The hydrogen bonds formed in the DES-CA complex are summarized in Table 3.

According to the data presented in Table 3 and the structural configuration shown in Figure 5c, DES1 is likely to interact with CA via four hydrogen bonds (O_32_-H_36_···Cl_28_, O_74_-H_75_···Cl_28_, O_72_-H_73_···Cl_28_, and O_37_-H_38_···O_79_) in conjunction with π-π conjugation interactions.

Furthermore, the binding energy (*ΔE*) was calculated via the following equation to quantitatively analyze the interaction strength between DES1 and CA.

Δ*E* = *E*_(DES1 − CA complex)_ − (*E*_DES1_ + *E*_CA_)

where *E*(_DES1 − CA complex_), *E*_DES1_, and *E*_CA_ represent the energies of the complex, DES1, and CA, respectively. The *ΔE* of DES1 with CA was calculated to be −25.641 kcal/mol. The negative value indicates a strong intermolecular interaction between DES1 and CA. A larger absolute value corresponds to a stronger interaction and a theoretically higher solubilizing capacity. For comparison, the *ΔE* values of CA with the conventional solvents water and ethanol were −5.277 kcal/mol and −8.946 kcal/mol, respectively. Therefore, the *ΔE* value of DES1 demonstrates that the hydrogen-bonding interaction between DES1 and CA is relatively strong, confirming that DES1 is an effective solvent for extracting CA from honeysuckle.

### 2.4. Comparison of the Proposed DES-MAE with the Conventional Methods

**Figure 6:**
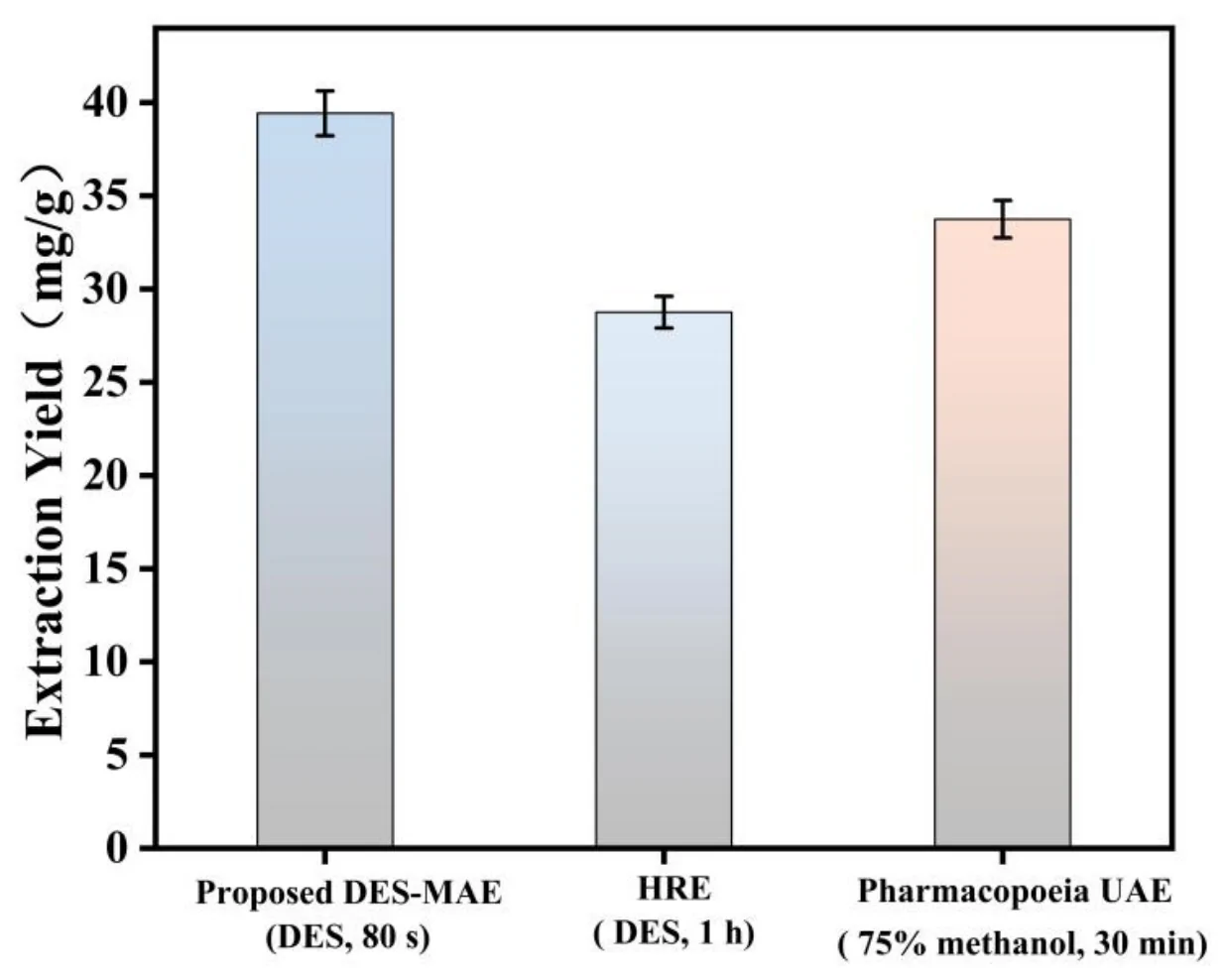
Comparison of the extraction yield of CA between the proposed DES-MAE and the conventional reference methods.

**Figure 7:**
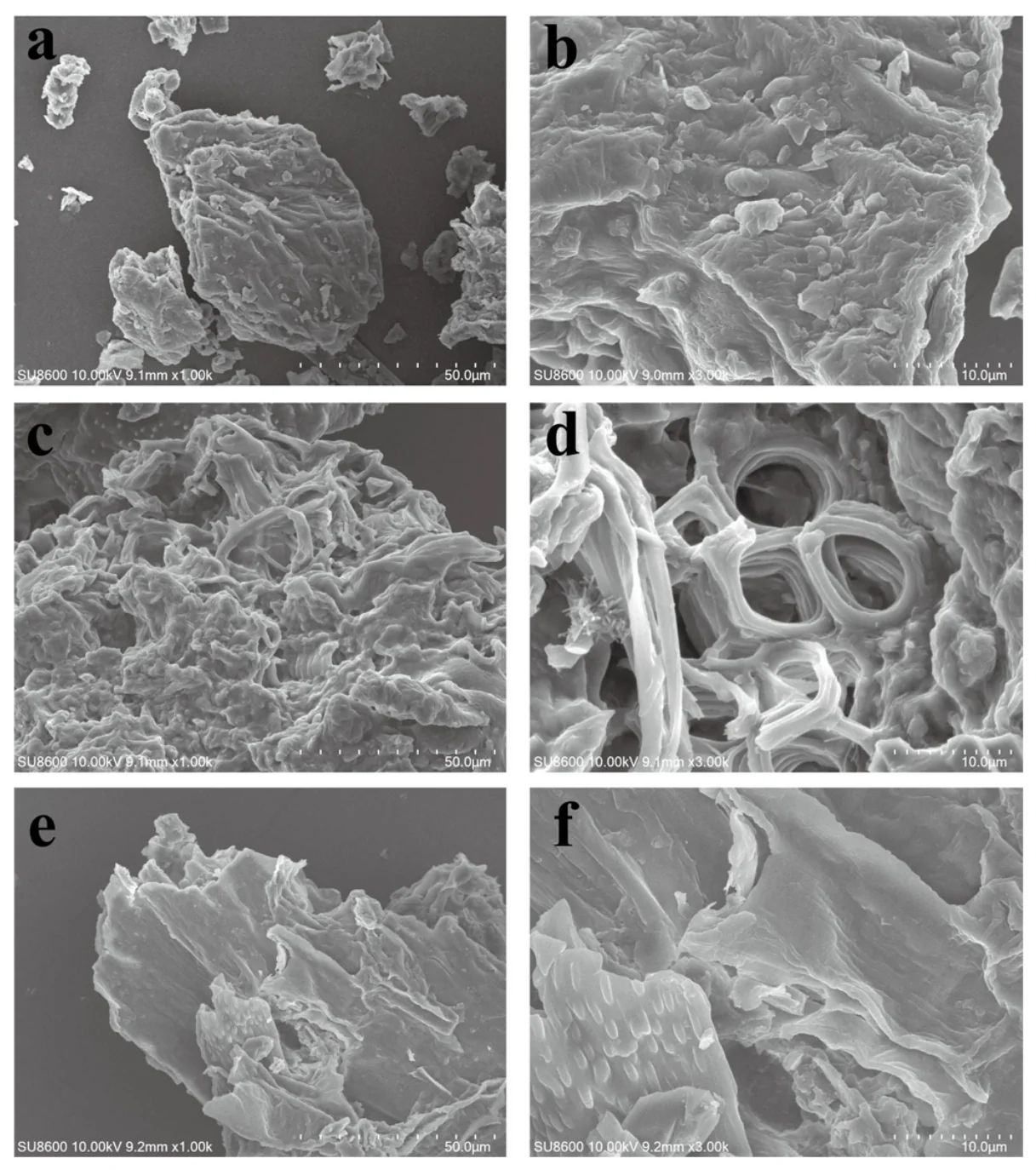
SEM images of honeysuckle powder untreated (**a**,**b**), treated with the proposed DES-MAE (**c**,**d**), and treated with the pharmacopoeia method (**e**,**f**).

To verify the advantages of combining DES and MAE in the proposed method, the extraction efficiencies of different methods for CA from honeysuckle were evaluated. The DES-MAE method established under optimal conditions was compared with optimized conventional methods, including DES-HRE and the pharmacopoeia method. The results shown in Figure 6 indicate that among the three extraction methods, the DES-HRE method exhibited the lowest extraction efficiency and required the longest extraction time. The pharmacopoeia method, which employed ultrasound assistance, shortened the extraction time to 30 min and improved the extraction efficiency. This may be attributed to the fact that ultrasound generates cavitation bubbles in the solvent. When these bubbles collapse rapidly, localized zones of high pressure and temperature are formed, thereby disrupting the plant cell walls. The DES-MAE method established in this study exhibited the highest extraction efficiency and drastically shortened the extraction time to 78 s. This may be ascribed to the fact that microwave irradiation can generate volumetric dielectric heating, which rapidly intensifies the disruption of plant cells and accelerates the mass transfer process, while the generated heat also increases the solubility of CA. To verify this hypothesis, SEM images of the surface of honeysuckle powder before and after ultrasound- and microwave-assisted extraction were captured and compared, and the results are shown in Figure 7a–f.

The untreated honeysuckle powder in Figure 7a,b exhibited a rough and intact surface. Figure 7c,d and Figure 7e,f present the SEM images obtained using the proposed method and the UAE method, respectively. As can be observed, under the action of the proposed method, the honeysuckle sample was thoroughly disrupted. Under the synergistic effect of DES dissolution and the rapid internal heating of MAE, the sample presented a more open and fragmented structure with ruptured plant cells, which is consistent with enhanced solvent penetration into the cellular compartments. Meanwhile, the short extraction duration may have helped minimize CA thermal degradation, thereby enhancing the extraction efficiency of CA. In contrast, the UAE method employed in the pharmacopoeia also caused certain damage to the honeysuckle structure, but to a lesser extent than the proposed method. Moreover, the pharmacopoeia method required a longer extraction time and utilized environmentally hazardous methanol as the extraction solvent. By contrast, DES is environmentally friendly. Therefore, the proposed method is considered an efficient and potentially sustainable extraction technique. Notably, even compared with previously reported methods, such as ethanol-based MAE [12] and DES-based UAE [10], the proposed method achieved the shortest extraction time and a high CA extraction efficiency, suggesting the potential advantages of combining DES and MAE under the tested conditions.

## 3. Materials and Methods

### 3.1. Materials

**Figure 8:**
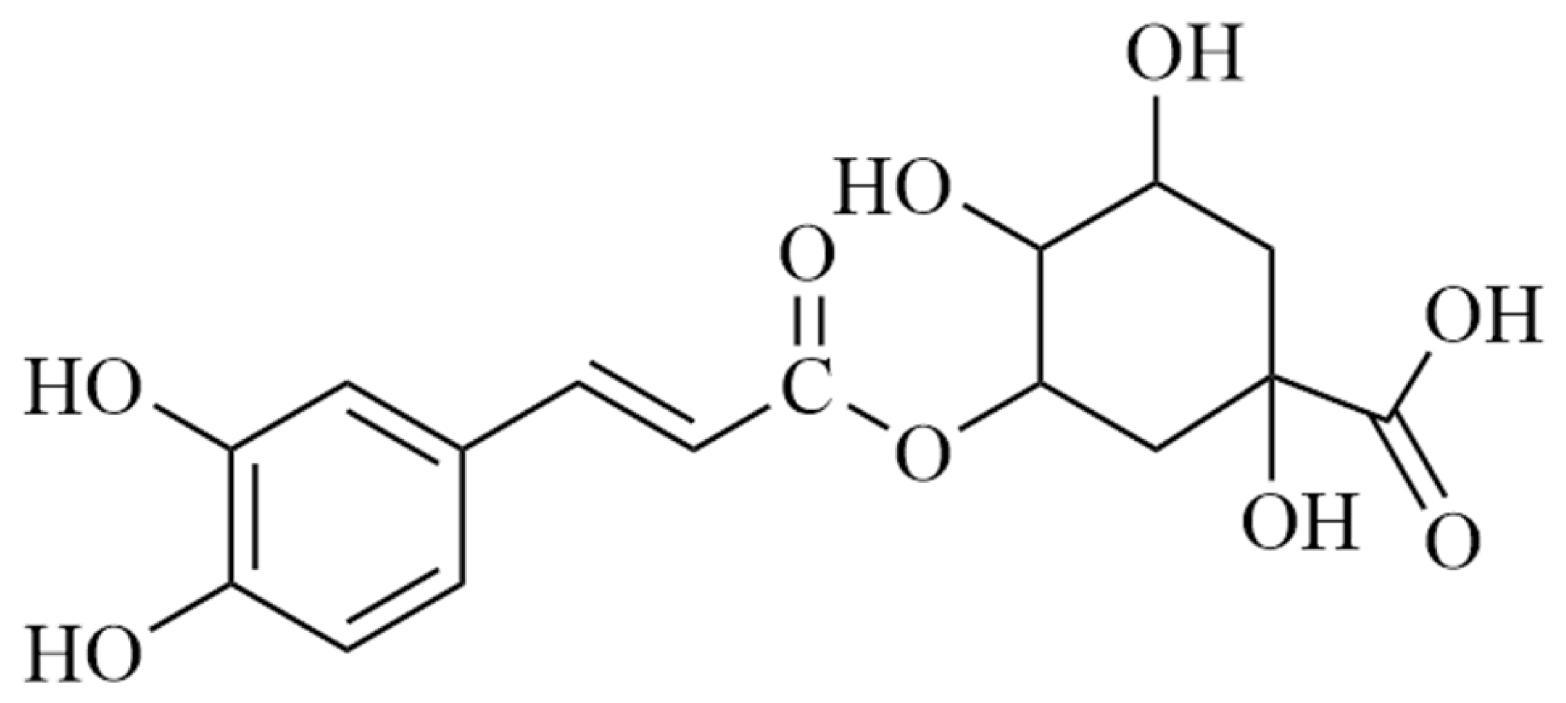
Chemical structure of chlorogenic acid (CA).

Chlorogenic acid (CA, ≥98%) was purchased from Aladdin Industrial Inc. (Shanghai, China). The chemical structure of CA is shown in Figure 8. Honeysuckle was purchased from Tongrentang Pharmacy (Xinxiang, China) and authenticated by a pharmacognosy expert from Henan Medical University. Methanol (HPLC grade) was obtained from Tedia Company (Fairfield, OH, USA). Benzyltrimethylammonium chloride, benzyltriethylammonium chloride, benzyltributylammonium chloride, tetrabutylammonium chloride, glycerol, ethylene glycol, and ethanol (all of analytical grade) were purchased from Sinopharm Chemical Reagent Co., Ltd. (Shanghai, China). Ultrapure water produced by a Milli-Q academic water-purification system (Merck Corporation, Molsheim, France) was used throughout the experiment. All other chemicals were at least of analytical reagent grade.

### 3.2. Synthesis of DESs

Based on our previous work and the relevant literature, four quaternary ammonium salts—benzyltrimethylammonium chloride, benzyltriethylammonium chloride, benzyltributylammonium chloride, and tetrabutylammonium chloride—were employed as HBAs, while glycerol and ethylene glycol served as HBDs. Each HBA was mixed with the corresponding HBD at a molar ratio of 1:1 in a round-bottom flask. Taking DES1 as an example, it was prepared by mixing 1.857 g (10 mmol) of benzyltrimethylammonium chloride with 0.621 g (10 mmol) of ethylene glycol. The mixture was magnetically stirred at 90 °C for 1 h until a clear, transparent solution was formed. Fourier-transform infrared spectroscopy (FT-IR) (model Vertex 70; Bruker Corporation, Billerica, MA, USA) was employed to characterize the synthesized DES1, and the results are presented in Figure S3. As shown in the spectrum, the broad absorption band at 3395 cm^−1^ in neat ethylene glycol (EG) is attributed to the stretching vibration of the hydroxyl groups. Upon the formation of DES1, this band shifted to a lower wavenumber of 3385 cm^−1^ with a broadening of the peak profile. In addition, the C-N stretching vibration of benzyltrimethylammonium chloride (TMBAC) exhibited a slight shift from 982 cm^−1^ to 975 cm^−1^ after DES formation, suggesting that the hydrogen-bonding interaction between the chloride anion and EG induced a subtle change in the electronic environment of the quaternary ammonium cation. This observation, together with the significant red shift and broadening of the O-H stretching band of EG, further confirms the formation of a hydrogen-bonded deep eutectic solvent network, thereby verifying the successful synthesis of DES1. The residual water content of the as-synthesized DESs was verified by Karl Fischer titration to be negligible, and thus no correction was required for the subsequent preparation of DES water mixtures. All DESs were stored in sealed amber glass vials and kept in a desiccator at room temperature (25 °C) in the dark prior to use. The compositions and abbreviations of the prepared DESs are listed in Table 4.

### 3.3. DES Extraction Coupled with MAE

The honeysuckle material was placed in a vacuum drying oven (Model 1400E, VWR Scientific Products, West Chester, PA, USA) and held at 40 °C until a constant weight was achieved. The dried material was then milled into powder using a pulverizer and sieved through a 40-mesh screen. The homogenized powder was collected and stored for subsequent extraction procedures.

In the DES-MAE procedure, 50 mg of honeysuckle powder was accurately weighed and transferred into a 25 mL conical flask, followed by the addition of 3 mL of DES and 2 mL of ultrapure water. The mixture was thoroughly vortexed, and the flask was sealed before being placed into a domestic microwave oven (model G80F23DCSL-F7(R0); Guangdong Galanz Electric Appliance Co., Ltd., Foshan, China). Extraction was carried out at ambient pressure without any pressurized system under varying microwave powers and irradiation durations. After the extract was allowed to cool down to room temperature, it was centrifuged at 10,000 rpm for 5 min using a Heraeus Multifuge X1R high-speed refrigerated centrifuge (Thermo Scientific, Waltham, MA, USA). The resulting supernatant was diluted 50 times with ultrapure water, filtered through a 0.22 μm membrane, and then subjected to HPLC analysis. The extraction yield of CA was calculated according to the following equation:

Yield (mg/g) *=* C × V × N/W × 1000

where C is the CA concentration in the diluted extract determined by HPLC (μg/mL), V represents the total volume of the extraction solution (mL), N indicates the dilution factor, and W is the mass of the honeysuckle sample (g). All extraction experiments were performed in triplicate (*n* = 3 independent samples) and conducted in a randomized order to minimize systematic bias. Each extract was injected in triplicate. All results are expressed as mean ± standard deviation (SD).

### 3.4. Conventional Reference Extraction Procedures

To validate the performance of the DES-MAE method, the Chinese Pharmacopoeia 2020 Edition method and HRE were employed as reference methods.

According to the Chinese Pharmacopoeia method, an accurately weighed amount (500 mg) of honeysuckle powder was transferred into a stoppered conical flask, and 50 mL of 75% methanol was added as the extraction solvent. The flask was sealed, weighed, and subjected to ultrasonic extraction (500 W, 40 kHz) using a KQ-500V ultrasonic generator from Kunshan Ultrasonic Instrument Co., Ltd. (Kunshan, China) for 30 min. Upon cooling, the weight loss was compensated with 75% methanol. The mixture was filtered through a 0.22 μm aqueous membrane filter, and the filtrate was diluted 5-fold with ultrapure water before HPLC analysis.

The HRE procedure was the same as that reported in the literature [29]. First, 1 g of honeysuckle powder was weighed into a 50 mL vial, and 20 g of DES (30% water content, *w*/*w*) was added. After sealing and vigorous mixing, the extraction of CA was performed at 70 °C for 60 min in a water bath with magnetic stirring (100 rpm). The resulting mixture was centrifuged at 9000 rpm for 30 min. The supernatant was collected and analyzed by HPLC.

### 3.5. Quantification of CA with HPLC

**Figure 9:**
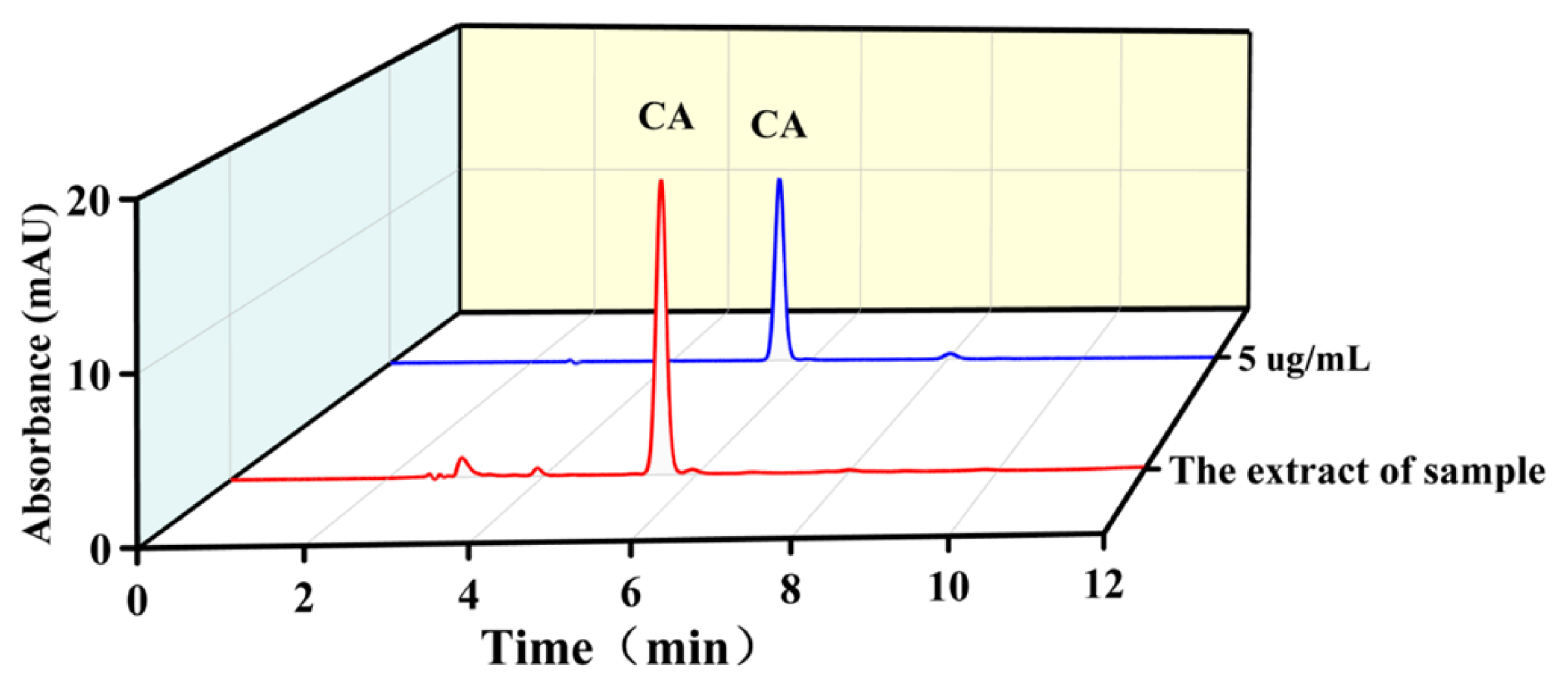
HPLC chromatogram of 5 µg/mL CA standard solution and the extract of the sample.

The content of CA was determined using an Agilent 1200 HPLC system (Agilent Technologies, Santa Clara, CA, USA). Sample separation was performed on a ZORBAX Eclipse XDB-C18 column (4.6 mm × 250 mm, 5 µm; Agilent Technologies, Santa Clara, CA, USA) at a column temperature of 35 °C, with an injection volume of 10 µL and a detection wavelength of 327 nm. The mobile phase consisted of a 0.2% phosphoric acid aqueous solution and methanol (7:3, *v*/*v*) at a flow rate of 1.0 mL/min. Each sample was injected in triplicate. Under the optimized conditions, the established analytical method was systematically validated. The calibration curve was constructed by plotting the peak area against the concentration of CA. Good linearity was observed over the concentration range of 0.1–10 µg/mL, with the regression equation Y = 24.583X − 0.9737 and a correlation coefficient (R^2^) of 0.9999. The limit of detection (LOD, S/N = 3) and limit of quantification (LOQ, S/N = 10) were 0.015 and 0.082 µg/mL, respectively. The intra-day and inter-day precisions were evaluated by six replicate measurements within one day and over three consecutive days, yielding relative standard deviations (RSDs) of 2.6% and 4.3%, respectively. The recovery test was performed by spiking CA standards at three concentration levels into the sample matrix, and the recoveries ranged from 98.6% to 101.8% with RSDs below 5.1%. In addition, the sample stability was confirmed for 24 h at 4 °C (RSD < 1.3%). Representative chromatograms of CA from the standard solution and the honeysuckle extract obtained with DES-MAE are shown in Figure 9.

### 3.6. Scanning Electron Microscopy (SEM) Analysis

The microstructural features of honeysuckle samples prior to and following extraction via various approaches were examined using scanning electron microscopy (SEM). Both untreated and extracted samples were visualized with an SU-8600 scanning electron microscope (Hitachi High-Tech Co., Ltd., Tokyo, Japan) under secondary electron imaging conditions with an accelerating voltage of 10 kV. Before SEM observation, a thin platinum coating was deposited onto all samples by sputtering at ambient temperature.

### 3.7. Optimization of the CA Extraction

Given that both the DES type and water content strongly affect CA extraction from honeysuckle, eight DESs with varying water contents were synthesized and screened via microwave-assisted extraction, and the most efficient one was selected for subsequent optimization. The factors—irradiation time, solid–liquid ratio, and microwave power—that affect extraction efficiency were studied through single-factor experiments. All single-factor experiments were performed in triplicate. Taking these results as a foundation, the optimization of the CA extraction procedure was further performed by adopting a three-factor, three-level Box–Behnken design under response surface methodology, with Design-Expert 13 serving as the statistical tool. The comprehensive arrangement of factors and levels is presented in Table 5.

### 3.8. Density Functional Theory (DFT) Calculations

All density functional theory (DFT) calculations were performed using the Gaussian 16 software. Molecular models of the DES, consisting of TMBAC and EG, as well as its complexes, were constructed in GaussView. The monomer configurations of HBD and HBA were first optimized at the B3LYP/6-311G(d,p) level, and the structures with the lowest energies were selected for electrostatic potential (ESP) calculations. Based on the charge distribution, the possible binding sites were predicted, and the initial structures of the DES-CA complexes were constructed. The structures of the solvents and complexes were further refined at the B3LYP/6-311++G(d,p) level, with particular attention paid to fine-tuning the atoms involved in hydrogen-bonding interactions, and the most stable conformers with the lowest energies were selected. The DFT-D3 dispersion correction was incorporated to accurately describe hydrogen bonding and weak van der Waals interactions. All optimized geometries were verified by vibrational frequency calculations to have no imaginary frequencies, confirming them as local minima on the potential energy surface.

For charge treatment, the default Mulliken population analysis as implemented in Gaussian 16 was used, and no additional charge scheme was explicitly specified. The DFT-D3 dispersion correction with Becke–Johnson damping was applied to accurately describe hydrogen bonding and van der Waals interactions. Multiple initial conformations were constructed and optimized, and the lowest-energy conformer without any imaginary frequency was selected for each system. The interaction energies were calculated using the optimized geometries and corrected for basis set superposition error (BSSE) via the counterpoise method (counterpoise = 2). All calculations were performed in the gas phase; explicit solvent effects were not modeled because the primary objective was to elucidate the intrinsic intermolecular interactions between DES1 and CA at the molecular level.

### 3.9. Statistical Analysis

All experimental data were statistically analyzed with Design-Expert^®^ software (ver. 13, Stat-Ease Inc., Minneapolis, MN, USA). Model adequacy was assessed via analysis of variance (ANOVA), and differences were considered statistically significant at the 95.0% confidence level according to the total error criterion. Response surface plots were generated using the same software to elucidate the correlations between the independent variables and the response.

## 4. Conclusions

In this study, an efficient and rapid DES-MAE method was successfully established for the efficient extraction of CA from honeysuckle. Among the eight synthesized DESs, a benzyltrimethylammonium chloride–ethylene glycol mixture (DES1) with 40% water content was identified as the optimal extraction solvent, owing to its low viscosity, appropriate polarity, and strong hydrogen-bonding interaction with CA. The extraction parameters were systematically optimized through single-factor experiments and Box–Behnken design-based response surface methodology. The established quadratic model demonstrated excellent goodness of fit and predictive capability (*R*^2^ = 0.9879, Adj *R*^2^ = 0.9724, Pred *R*^2^ = 0.8650), with all quadratic terms reaching high significance (*p* < 0.001). Under the optimized conditions (microwave power of 640 W, irradiation time of 78 s, solid-to-liquid ratio of 9.35 mg/mL), the CA extraction yield reached 39.431 ± 0.068 mg/g (relative deviation 1.1%), in excellent agreement with the predicted value of 39.851 mg/g. Mechanistic investigations via DFT calculations and SEM analysis provided molecular-level insights into the superior extraction performance. The DES1-CA complex was stabilized by four hydrogen bonds and π-π stacking interactions, with a binding energy (−25.64 kcal/mol) nearly 3–5 times higher than those of conventional solvents (water and ethanol), confirming the strong solvation capacity of DES1. SEM images revealed that the synergistic action of DES dissolution and microwave volumetric dielectric heating thoroughly disrupted the plant cell wall, producing an open and fragmented microstructure that facilitated solvent penetration into cellular compartments, while the ultra-short extraction time (78 s) effectively avoided CA thermal degradation. Compared with DES-based heat-reflux extraction (60 min) and the pharmacopoeial ultrasound-assisted method (30 min, employing toxic methanol), the DES-MAE method established in this study achieved a high extraction yield with the shortest extraction time and an environmentally benign solvent. Therefore, this method provides a promising, efficient and sustainable approach for the extraction of bioactive constituents from traditional Chinese medicinal materials, while the integrated experimental–computational strategy offers a valuable reference for the design and screening of novel DESs for targeted extraction applications.

## Figures and Tables

**Table 1:** Three factors and three levels, the Box–Behnken design, and the results.

Run	A: Microwave Power (W)	B: Irradiation Time (s)	C: Solid–Liquid Ratio (mg/mL)	Extraction Yield (mg/g)
1	480	80	5	27.013
2	640	80	10	39.790
3	640	80	10	38.264
4	800	100	10	18.894
5	640	60	5	31.028
6	640	100	15	25.670
7	480	60	10	24.630
8	800	80	15	24.477
9	480	100	10	24.270
10	800	80	5	26.126
11	480	80	15	26.552
12	640	60	15	23.374
13	640	100	5	25.894
14	640	80	10	39.780
15	640	80	10	40.793
16	800	60	10	25.533
17	640	80	10	39.837

**Table 2:** Analysis of variance (ANOVA) of the Box–Behnken design.

Source	Sum of Squares	Degree of Freedom	Mean Square	*F*-Value	*p*-Value	Significant
Model	811.19	9	90.13	63.73	0.00001	significant
A	6.91	1	6.91	4.886	0.06275	
B	12.10	1	12.09	8.553	0.0222	
C	12.48	1	12.47	8.822	0.0208	
AB	9.86	1	9.85	6.969	0.03343	
AC	0.3528	1	0.3528	0.2495	0.6328	
BC	13.80	1	13.80	9.761	0.01674	
A^2^	297.48	1	297.48	210.36	0.000002	
B^2^	266.50	1	266.50	188.45	0.000003	
C^2^	115.85	1	115.85	81.92	0.00004	
Residual	9.90	7	1.41			
Lack of Fit	6.61	3	2.202	2.678	0.1826	not significant
Pure Error	3.29	4	0.8226			
Cor Total	821.09	16				
*R*^2^ = 0.9879						
*C.V.*% = 4.03						

**Table 3:** Hydrogen bonds and their properties of DES1 and DES1-CA complexes.

Structure	Hydrogen Bonds	Bond Length (Å)	Bond Angle (°)
DES1	O_32_-H_36_···Cl_28_	2.17	175.0
DES1-CA Complexes	O_32_-H_36_···Cl_28_	2.26	171.8
	O_74_-H_75_···Cl_28_	2.24	156.3
	O_72_-H_73_···Cl_28_	2.27	157.9
	O_37_-H_38_···O_79_	2.07	149.4

**Table 4:** The composition of the DESs and their abbreviations.

Abbreviation	HBA	HBD	Molar Ratio
DES1	benzyltrimethylammonium chloride	ethylene glycol	1:1
DES2	benzyltrimethylammonium chloride	glycerol	1:1
DES3	benzyltriethylammonium chloride	ethylene glycol	1:1
DES4	benzyltriethylammonium chloride	glycerol	1:1
DES5	benzyltributylammonium chloride	ethylene glycol	1:1
DES6	benzyltributylammonium chloride	glycerol	1:1
DES7	tetrabutylammonium chloride	ethylene glycol	1:1
DES8	tetrabutylammonium chloride	glycerol	1:1

**Table 5:** Factors and levels in the response surface design.

Factor	Level		
	−1	0	1
A: Microwave power (W)	480	640	800
B: Irradiation time (s)	60	80	100
C: Solid–liquid ratio (mg/mL)	5	10	15

## Data Availability

The original contributions presented in this study are included in the article/Supplementary Material. Further inquiries can be directed to the corresponding authors.

## Supplementary Materials

The following supporting information can be downloaded at https://www.mdpi.com/article/10.3390/molecules31193545/s1, Figure S1: Normal probability of residuals; Figure S2: The residuals vs. predicted; Figure S3: FT-IR spectra of ethylene glycol (EG), benzyltrimethylammonium chloride (TMBAC), and DES1.

## Abbreviations

The following abbreviations are used in this manuscript:

CA	chlorogenic acid
DES	deep eutectic solvent
MAE	microwave-assisted extraction
DFT	density functional theory
RSM	response surface methodology
HRE	heat-reflux extraction
HBA	hydrogen-bond acceptor
HBD	hydrogen-bond donor
HPLC	high-performance liquid chromatography
BBD	Box–Behnken Design
SEM	scanning electron microscopy
TMBAC	benzyltrimethylammonium chloride
TEBAC	benzyltriethylammonium chloride
TBBAC	benzyltributylammonium chloride
TBAC	tetrabutylammonium chloride
EG	ethylene glycol
Gly	glycerol
ESP	electrostatic potential
