# Supplementary material for: Rapid and Efficient Extraction of Chlorogenic Acid from Honeysuckle Using Microwave-Assisted Deep Eutectic Solvents: Process Optimization and Mechanistic Investigation via Integration of Experiments and Density Functional Theory Calculations
Source: Molecules. 2026 Oct 4;31(19):3545. doi: 10.3390/molecules31193545 (PMC13666877; doi:10.3390/molecules31193545)
Supplement: Supplementary file 1 [file molecules-31-03545-s001.zip › molecules-4546288-supplementary.pdf]

Supplementary Information

Rapid and Efficient Extraction of Chlorogenic Acid from Honeysuckle Using Microwave-Assisted Deep Eutectic Solvents: Process Optimization and Mechanistic Investigation via Integration of Experiments and Density Functional Theory Calculations

Hongwei Wu [1,*], Ruixin Chen [1], Hu Feng [1], Ningfei Liu [1], Yongli Shi [2], Feng Wang [3] and Tiancheng Mu [4,*]

[1] School of Basic Medical Sciences, Henan Medical University, Xinxiang 453003, China; 18338918237@163.com (R.C.); fh20190617@163.com (H.F.); 13849924802@163.com (N.L.)

[2] College of Pharmacy, Henan Medical University, Xinxiang 453003, China; shiyongli2005@163.com

[3] Henan Key Laboratory of Tissue Regeneration, School of Basic Medical Sciences, Henan Medical University, Xinxiang 453003, China; fengw@xxmu.edu.cn

[4] School of Chemistry and Life Resources, Renmin University of China, Beijing 100872, China

* Correspondence: 011026@xxmu.edu.cn (H.W.); tcmu@ruc.edu.cn (T.M.); Tel.: +86-373-302-9913 (H.W.)

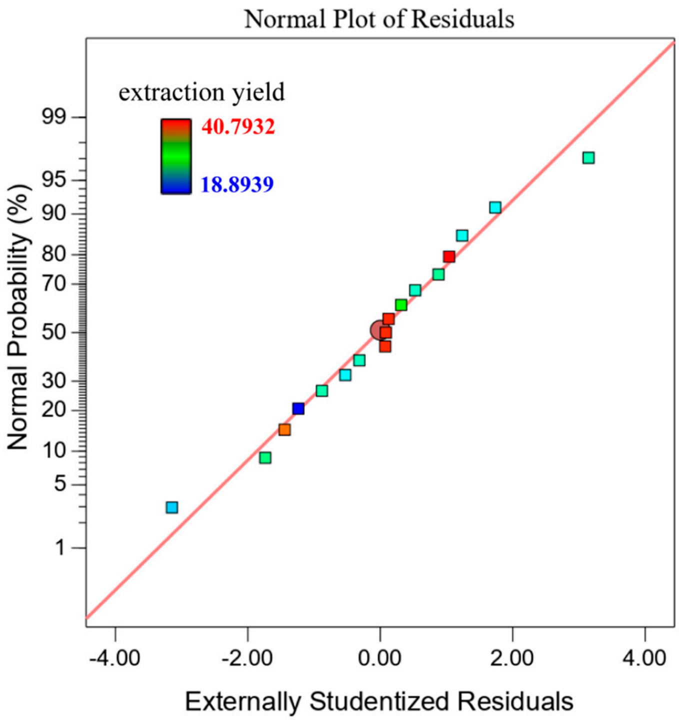

Figure S1. Normal probability of residuals.

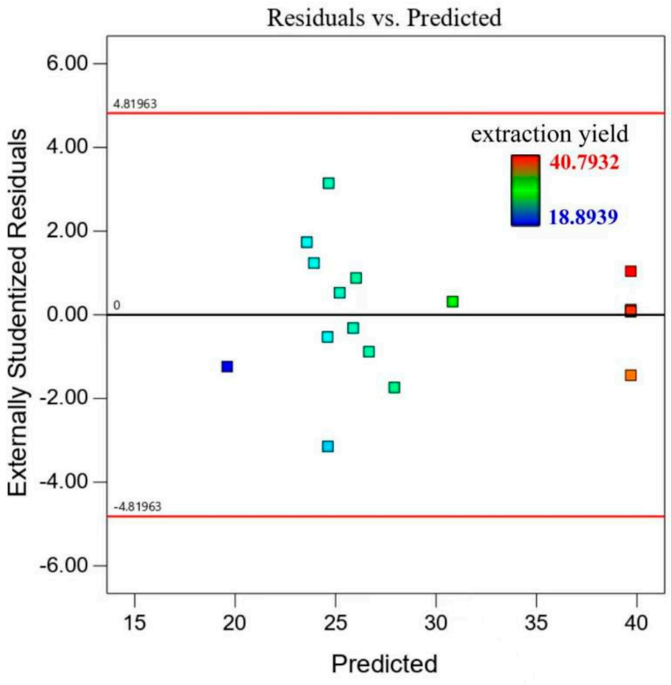

Figure S2. The residuals vs. predicted.

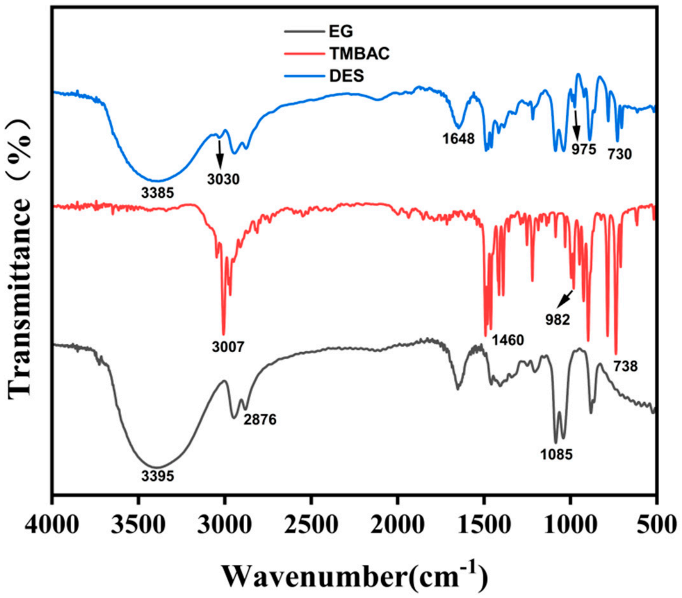

Figure S3. FT-IR spectra of ethylene glycol (EG), benzyltrimethylammonium chloride (TMBAC), and DES1.
